# Innovative pressure environment combining hydrostatic pressure gradient and mechanical compression for structural investigations of nanoporous soft films

**DOI:** 10.1107/S1600577522005914

**Published:** 2022-06-23

**Authors:** Julie Wolanin, Jérôme Giraud, Isabelle Morfin, Anne-Laure Rollet, Laurent Michot, Marie Plazanet

**Affiliations:** a Univ. Grenoble Alpes, CNRS, LIPhy, 38000 Grenoble, France; b Sorbonne Université, CNRS, Laboratoire Physico-chimie des Electrolytes et Nanosystèmes Interfaciaux, PHENIX, F-75005 Paris, France

**Keywords:** high-pressure cell, soft matter, porous materials, cellulose, X-ray scattering

## Abstract

The development of a new sample environment enabling X-ray scattering measurements at small and large angles under mechanical compression and hydraulic flow is presented. The cell, which is adapted for moderate pressures, includes beryllium windows, and allows applying simultaneously a compressive pressure up to 2.5 kbar in the perpendicular direction to the flow and either a hydrostatic pressure up to 300 bar or a pressure gradient of the same amplitude. The development of high-pressure devices for synchrotron experiments is relevant for many scientific fields in order to unveil details of a material’s structure under relevant conditions of stresses. In particular, mechanical constraints coupled to hydrostatic pressure or flow, leading to complex stress tensor and mechanical response, and therefore unexpected deformations (swelling and pore deformation), are poorly addressed. Here, first the design of the environment is described, and then its performance with measurements carried out on a regenerated cellulose membrane is demonstrated.

## Abstract 2

The innovative pressure cell described, suitable for X-ray measurements combining both hydraulic and compressive pressures, is well adapted for studying poromechanical coupling in soft environments.

## 1. Introduction

Water transport in soft porous membranes is crucial in many industrial processes such as ultrafiltration or reverse osmosis. Multiscale characterization of the membranes is needed for predicting water transport in such materials. Compared with the case of hard porous materials for which water transport has been widely investigated (Teixeira *et al.*, 1997 ▸; Mitra *et al.*, 2001 ▸; Coasne *et al.*, 2013 ▸; Briman *et al.*, 2012 ▸; Taschin *et al.*, 2013 ▸), much less work has been devoted to soft materials. This is likely related to the fact that, in soft matter, numerous additional effects are at play. Among them, one may quote surface heterogeneity (hydrophilicity/hydrophobicity heterogeneities of the confining surface), the influence of sorption phenomena, and the role of mechanical effects (both local and global host deformations) (Klein *et al.*, 2013 ▸; Ha & Kim, 2020 ▸). In any case, characterizing the structural evolution (swelling and pore deformation) of soft membranes under various water pressures and mechanical stresses represents an important issue (Rosti *et al.*, 2020 ▸; MacMinn *et al.*, 2016 ▸). To address this question, we here present the design of a mild-pressure cell suitable for investigating poromechanical coupling in soft porous materials using X-ray scattering. If various setups have been proposed, such as for example by Le Vay *et al.* (2020 ▸), Brooks *et al.* (2010 ▸) and Lehmkühler *et al.* (2019 ▸), to the best of our knowledge the present one has not been reported in the literature. The proposed pressure cell enables the simultaneous application of hydrostatic (or flowing) pressures up to 300 bar and of mechanical compression in the perpendicular direction up to ∼2.5 kbar. After describing the proposed pressure cell, we demonstrate its performance in presenting synchrotron measurements performed on a cellulosic membrane. The choice of this material was motivated by its well characterized structure under various conditions and its wide range of applications, including stress under flowing conditions.

## 2. Pressure X-ray cell

The sample environment is designed for allowing simultaneous application of hydraulic pressure and mechanical compression in the perpendicular direction. In order to collect small- and wide-angle X-ray scattering (SAXS and WAXS, respectively) data, a scattering angle of 20.56° is achieved. The body material is stainless steel to ensure negligible mechanical deformations, while beryllium windows provide a high transmission for the X-ray beam. The cell withstands a water pressure gradient up to 300 bar simultaneously to a compressive pressure up to ∼2.5 kbar.

### 2.1. Cell design

**Figure 1:**
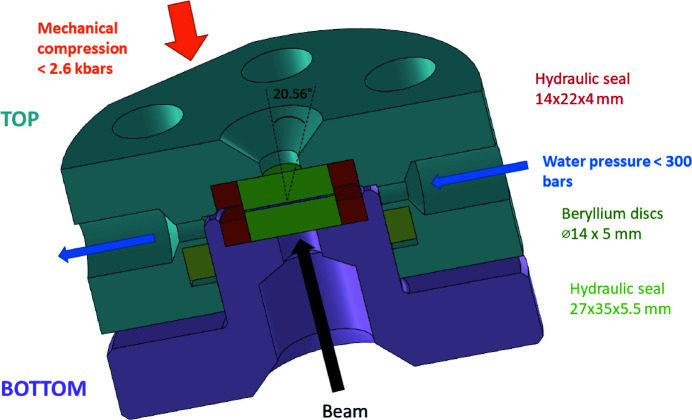
Sketch of the cell.

The cell frame’s design is presented in Fig. 1 ▸ and drawings are given in the supporting information. To ensure homogeneous pressure on the whole membrane while avoiding possible corrosion, we chose a stainless steel body. The top (containing the hydraulic path) and the bottom parts are assembled with six screws. The compression of the sample can be tuned by tightening the screws at different torque values. The relationship between the applied torque and the corresponding compressive pressure was calibrated on a similar cell with different dimensions as reported by Wolanin *et al.* (2021 ▸) and still applicable to the present cell. Moreover, finite-elements-based poromechanical simulations of the stress distribution in the sample presented in the same paper showed a very flat residual stress (in the case of hydrostatic pressure, *i.e.* no pressure gradient) beside deviation on the very edges of the samples. The measurement zone is therefore in a well defined stress region. The main advantage of such a design is that a linear relationship is ensured until a torque value of 5 N m (1 N m corresponds to ∼500 bar for this windows size). Above 5 N m, the pressure/load curve flattens. As shown in Fig. 1 ▸, the stainless steel body is hollowed for the beam path and indented on its way out to maximize the reachable scattering angle.

Water-tightness is achieved using hydraulic seals purchased from Elastotech (US 594, 12×22×4/4.5 around the windows and UTL 593, 27×35×5.5/6.5). A hand pump with spindle drive (609 28 00 from Top Industries) enables the application of the hydrostatic pressure, constantly monitored by a digital pressure sensor. The choice of this hydraulic seal limits the hydraulic pressure to 300 bar. However, we emphasize that a higher water pressure could be achieved with another kind of hydraulic seal.

Mechanical compression is therefore applied in a perpendicular direction with respect to the hydraulic flow. All measurements were performed at room temperature without further temperature control other than that of the experimental hutch.

### 2.2. Beryllium windows

The investigated sample is sandwiched between the two beryllium windows.

According to the literature, most high-pressure cells for X-ray studies are built using beryllium (Steinhart *et al.*, 1999 ▸; Winter, 2002 ▸; Pressl *et al.*, 1997 ▸) or diamond (Woenckhaus *et al.*, 2000 ▸; Quesada Cabrera *et al.*, 2011 ▸; McMahon, 2015 ▸; Shen & Mao, 2016 ▸) flat windows. In the present case, even though beryllium is more hazardous than diamond (toxic dust in case of breakage), we chose to use beryllium for both the input and output windows (incident and scattered beams) as it exhibits the best transmittance to tensile-strength ratio (Duesing *et al.*, 1996 ▸). Each window thickness was 5 mm in order to have an appropriate pressure resistance. The X-ray transmission at 15.7 keV of these two Be windows is 59%. For comparison, it would have been only 29% for the same thickness of diamond and 78% with 1 mm of diamond. The two beryllium windows were purchased from NGK Berylco France (with a purity >98.5%). In order to enlarge the scattering angle but still avoid window deformations under pressure, the chosen diameter (14 mm) is larger than those described previously (Winter, 2002 ▸; Pressl *et al.*, 1997 ▸) where pressures up to 3 kbar were used. Such a combination of body design and windows choice allows a maximum scattering angle of 20.56° to be reached.

### 2.3. SAXS and WAXS curves of the empty cell

**Figure 2:**
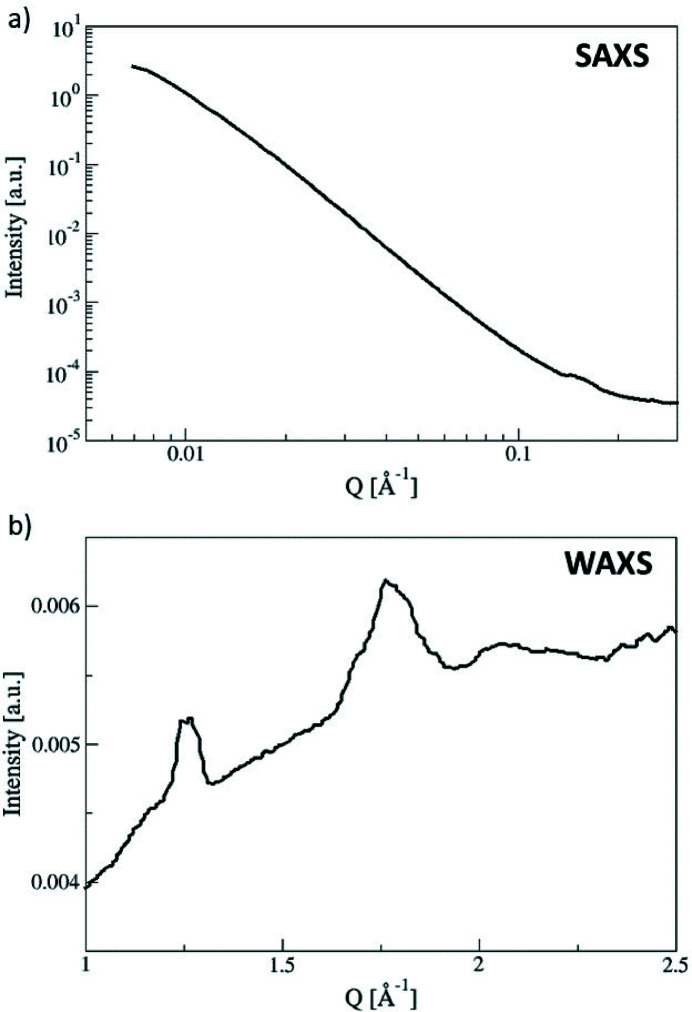
Empty cell scattering spectra: (*a*) SAXS and (*b*) WAXS data.

Synchrotron experiments were performed on the CRG-D2AM beamline (ESRF, Grenoble, France) with a wavelength of 0.775 Å (15.7 keV). We performed simultaneously WAXS and SAXS measurements using, respectively, the WOS and the D5 XPAD pixel detectors, the sample–detector distances being 9.4 and 112.6 cm. The *Q* calibrations of the scattering signals (*Q* = 4πsinθ/λ), where θ is half of the scattering angle and λ is the incident wavelength, were realized thanks to chromium oxide (Cr_2_O_3_) and silver behenate (AgBh) powder standards. WAXS and SAXS two-dimensional data were corrected by considering the detector distortion and the flat-field response of the detectors. D2AM programs based on *pyFAI* were used to perform data treatment and radial averaging. Scattered intensities were normalized by the transmission, and glassy carbon scattering was used to convert data into absolute units. The scattered intensities *I*(*Q*) versus *Q* were corrected by subtracting the signal of the beryllium empty cell. Measurements performed at different positions of the beryllium windows (*X* and *Z* axis ± 0.5 mm according to the center) were performed in order to check their homogeneities. The empty cell was only measured without hydrostatic pressure (no water) nor mechanical constraint. Since its subtraction from all the measurements did not create any particular feature in the data, we believe that its spectrum does not change with experimental conditions. The corresponding WAXS spectra display all the same scattering and two diffraction peaks that can be assigned to beryllium (Nabihah & Shanmugan, 2016 ▸; He *et al.*, 2016 ▸). This scattering [Fig. 2 ▸(*b*)] can be perfectly removed from the sample signals after subtraction.

The SAXS profile of the empty cell (beam centered on the Be disk) displayed in Fig. 2 ▸(*a*) exhibits high scattering at low *Q* that may prevent viable analysis in the *Q*-range below 0.05 Å^−1^ for low-scattering samples such as cellulose.

Radiation damage was not expected on D2AM with this setup (energy = 15.7 keV, transmission values around 60%) but has been verified by iteration of 30 s cellulose membrane measurements before the *in situ* experiments.

## 3. Application

### 3.1. Overview on the structural characterization of cellulose-based materials

Cellulose-based materials have attracted considerable interest over the past two decades owing to their potential for many industrial applications (cosmetics, food industry, pharmaceutical products, *etc*.), filling the increasing requirements for environmentally friendly and bio-compatible products. Thanks to the various valuable properties of cellulosic materials, considerable research has been performed in order to understand their structure–property relationships (Quesada Cabrera *et al.*, 2011 ▸; Poletto *et al.*, 2013 ▸). The macroscopic properties of cellulose are linked to its particular structure, comprising repeating glucose units yielding numerous architectures. Moreover, thanks to its advantageous mechanical properties (high mechanical strength and elastic modulus values) that can be tuned by chemical modifications, cellulose is used in diverse fields. Most appealing cellulose properties arise from many parameters such as inter- and intra-molecular hydrogen bonds, chain lengths and degree of crystallinity (Poletto *et al.*, 2013 ▸).

From a structural point of view, cellulose is a semi-crystalline polymer made of ringed glucose molecules. Three hydroxyl groups are present per repeating unit favoring the formation of hydrogen bonds between cellulose chains. The relative stability of cellulose and the cellulose fibrils stiffness (aggregation of many cellulose chains) is directly promoted by the intra- and inter-chain hydrogen bonds together with van der Waals interactions. Both highly ordered crystalline structure and amorphous regions can be found in cellulose fibrils at the nanoscale [for instance, cotton fibers have a degree of crystallinity of ∼40% (Quesada Cabrera *et al.*, 2011 ▸)]. The mechanical properties are directly related to the ratio between amorphous and crystalline regions, as well as to the properties of both individual regions.

Crystallographic studies on various cellulosic materials have been reported in the literature (Howell, 2008 ▸; Liu *et al.*, 2015 ▸, 2020 ▸; Håkansson *et al.*, 2014 ▸; Gubitosi *et al.*, 2017 ▸; Garvey *et al.*, 2005 ▸; Bates *et al.*, 2006 ▸; Jiang *et al.*, 2012 ▸). There are four different polymorphs of cellulose: cellulose I, II, III, and IV. Cellulose I (native cellulose) is the natural form of cellulose while cellulose II (regenerated cellulose) is made from dissolved cellulose I. It yields reconstituted fibers that can be useful for specific applications (textile fiber or papermaking industry for instance). Cellulose III and IV are amorphous and obtained by treatment of cellulose (I or II) and III, respectively. Within the framework of this study based on SAXS/WAXS measurements, only crystalline cellulose I and II will be considered.

Table 1 ▸ gathers the peak positions and their crystallographic assignation as reported in the literature for cellulose I and II. There are clear differences on the diffraction patterns for the two cellulose classes. Type I (native cellulose) displays a higher crystallization degree than Type II (regenerated cellulose). Furthermore, a broad halo linked to an amorphous contribution can also be observed. Ergo, cellulose can be treated as a two-phase system consisting of both disordered and highly ordered regions (Garvey *et al.*, 2005 ▸).

In this paper, we investigated a regenerated cellulose sample (cellulose II). According to Table 1 ▸, the typical regenerated cellulose structure displays two main features: a broad reflection of the (110) and (020) planes around 1.45 Å^−1^ and a peak around 0.9 Å^−1^ corresponding to the 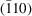 plane.

### 3.2. Experimental data

Regenerated cellulose acetate membranes were purchased from Merck Millipore and used as received. They have the form of ultrafiltration disks made of different porosity layers including a membrane NMWL (nominal molecular weight limit) of 1 kDa (corresponding to nanometric pores size). Cellulose acetate materials are industrially synthesized and can be used in a wide range of applications such as textiles, composites or polymeric membranes.

**Figure 3:**
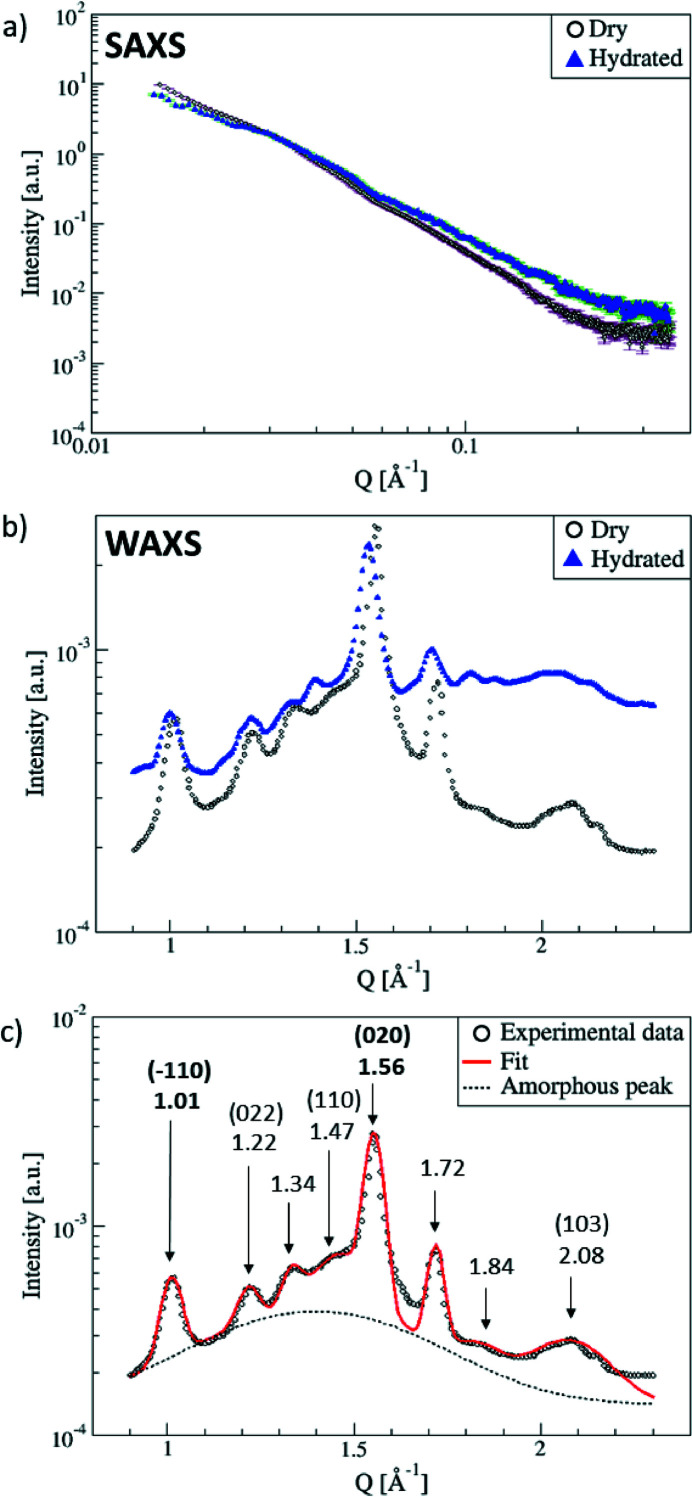
(*a*) SAXS and (*b*) WAXS spectra obtained for the dry and hydrated cellulose membranes measured with a standard holder (dry) and in the pressure cell (hydrated). Errors bars for the WAXS measurements are smaller than the symbols. (*c*) The reflection peaks of the dry sample were fitted with Gaussian functions and an amorphous peak was also added as a background contribution. The (*hkl*) values of the two peaks further analyzed are shown in bold.

The SAXS and WAXS measurements carried out on the dry and hydrated membranes are shown after empty cell subtraction in Fig. 3 ▸. Reliable signals are obtained in both the SAXS and WAXS regions, which validates the design of our setup. The SAXS signals of the hydrated membrane under various conditions of compressive stress and water pressure, presented in Fig. S3 of the supporting information, appear higher than the dry one. This confirms the presence of water in the cell. No significant difference is visible under the different conditions however. This may be linked to either the pore size distribution being out of the SAXS window or intrinsic stiffness of the cellulose. As a matter of comparison, we point out that variations in the SAXS signals of cellulose aerogels upon compression have been reported in the literature (Rennhofer *et al.*, 2019 ▸) at smaller *Q* (< 0.1 nm^−1^), outside the range of our analyzable *Q*-range. Still, the hydrated samples intensity is higher for *Q* > 0.05 Å^−1^, which confirms that water has swollen the cellulose membrane. We still point out that, with our pressure cell, in the analyzable SAXS *Q*-range, *i.e.* for *Q* higher than 0.05 Å^−1^, valuable information can be obtained using another sample such as Nafion for instance (see Figs. S4 and S5 of the supporting information). Thus, in the following, we will focus only on WAXS spectra.

The WAXS pattern of the dry regenerated cellulose membrane is presented in the middle panel of Fig. 3 ▸. It exhibits characteristic peaks of the crystalline regions of cellulose II, on top of an amorphous contribution. Upon hydration, a broad contribution appears underneath the dry membrane signal [Fig. 3 ▸(*b*)].

Based on the literature summarized in Table 1 ▸, the following peak assignment is proposed: 1.01 Å^−1^ for ( 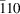 ), 1.22 Å^−1^ for (022), 1.47 Å^−1^ for (110), 1.55 Å^−1^ for (020), and 2.08 Å^−1^ for (103).

**Figure 4:**
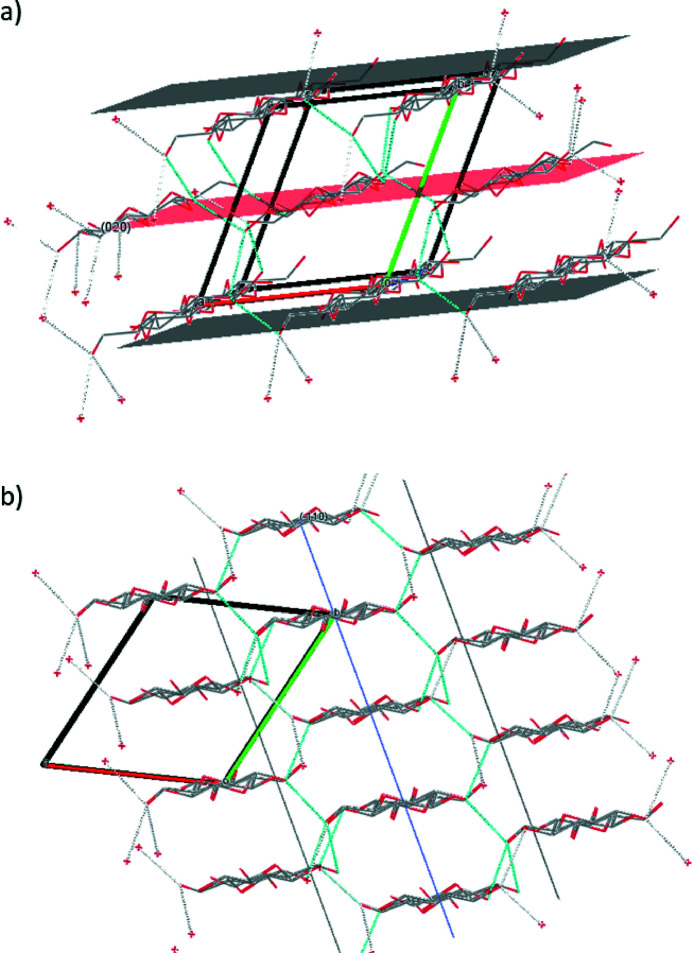
View of the structure of cellulose and the crystallographic planes: (*a*) for (020) and (*b*) for ( 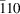 ). Structural data from French (2014 ▸).

The two peaks at 1.01 Å^−1^ and 1.55 Å^−1^ exhibit the highest intensities and will be discussed in the following. The crystallographic structure is represented in Fig. 4 ▸ together with these two particular crystallographic planes. The ( 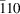 ) Bragg peak will be related to structural compression within the plane of the cellulose chains, while the (020) peak is characteristic of the compression between chain planes. Note that hydrogen bonds link the sugar units in a zigzag scheme, leading to interaction within the (020) plane and parallel to the ( 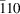 ) planes.

The cellulose membrane placed in our setup was subjected to water pressures up to 300 bar and mechanical compressions up to about 2.6 kbar (torque value of 8 N m). We monitored the associated structural changes. The empty cell background was subtracted to remove background and beryllium Bragg peaks.

**Figure 5:**
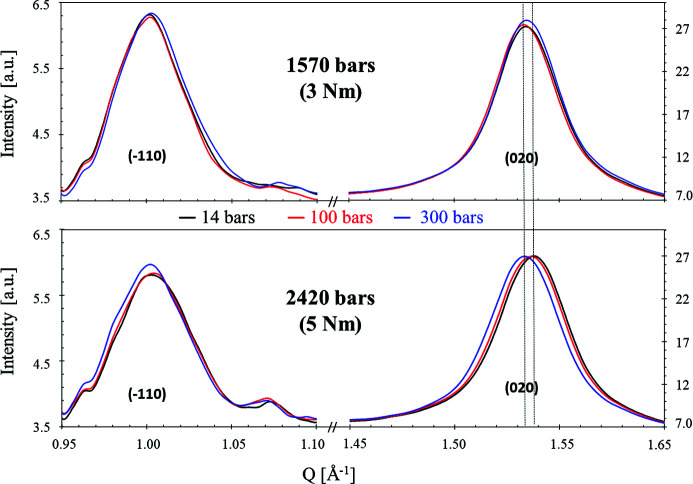
Influence of the hydrostatic pressure at fixed compressive pressure (torque values of 3 N m and 5 N m). The vertical lines are a guide to the eye to directly observe the effect of pressure on membrane swelling.

We first present in Fig. 5 ▸ the influence of the hydrostatic pressure at two constant compression levels, 3 and 5 N m (1570 bar and 2420 bar, respectively). At 3 N m, whatever the water pressure, no valuable changes are observed on both peaks excepted a tiny shift of the peaks toward the high-*Q* region, that could be due to a larger amount of water in the sample. At 5 N m, compared with 3 N m, the (020) peak is slightly shifted to higher *Q* for low water pressure (14 and 100 bar), revealing a tiny contraction of the planes (see the vertical lines, highlighting this observation). Once the water pressure reaches 300 bar, the peak shifts back to its initial position, which shows that water pressure compensates for the effect of compressive stress. A similar trend, with less pronounced variations, might occur on the ( 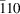 ) peak.

**Figure 6:**
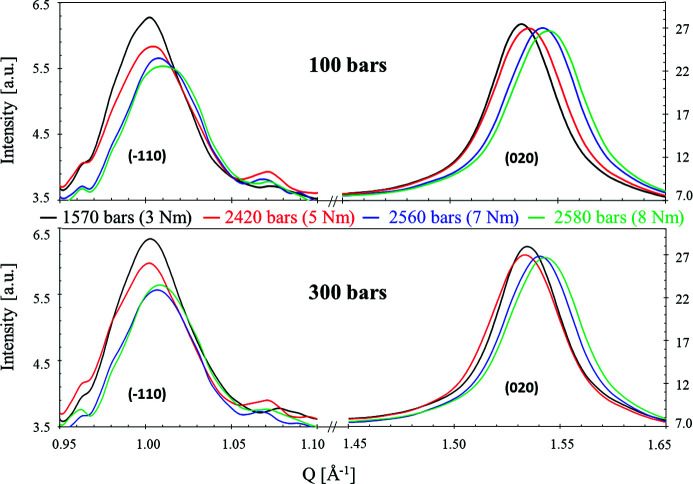
Influence of the compressive pressure at fixed hydrostatic pressure (100 bar and 300 bar).

Secondly, the influence of compressive force at two constant water pressures, 100 and 300 bar, is shown in Fig. 6 ▸. Significant shifts toward higher *Q* occur for both peaks and water pressures. But we also observe a different behavior for the two peaks: while the position, intensity and width are affected for the ( 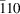 ) peak, only the position seems to be modified for the (020) peak. This observation suggests an increase of structural disorder with the compression (different arrangement of the cellulose chains within the fibers). The shift of the position of the (020) peak is essentially related to the variation of the distance between the planar sheets (see Fig. 4 ▸). It must be pointed out that torque tightening values of 3 N m, 5 N m, 7 N m and 8 N m correspond to about 1570 bar, 2420 bar, 2560 bar and 2580 bar, respectively (Wolanin *et al.*, 2021 ▸), *i.e.* to much stronger stresses with mechanical pressure than those produced with hydraulic pressure. The largest differences are observed between 5 and 7 N m. Small differences are observed between 7 and 8 N m, according to the small effective compressive pressure (2560 and 2580 bar). We also observe a different behavior for the two peaks: while the ( 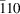 ) peak varies rather linearly with the applied force, the peak at (020) exhibits a larger variation between 5 and 7 N m than between 3 and 5 N m, indicating that each direction of compression does not have the same mechanical response.

Moreover, we observe that, whatever the conditions, the main differences in the intensity appear on the peak at 1.01 Å^−1^. This suggests an anisotropy of the compression module due to an easier distortion of the structure when compressing the planes of sugar units [plane ( 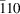 )] than when bringing the vertical alignments closer [plane (020)].

In order to model X-ray peak shapes and extract quantitative information, the two peaks have been fitted with Gaussian functions. A flat background, identical for all the measurements, has been added in order to make a comparison between the different tested conditions.

**Figure 7:**
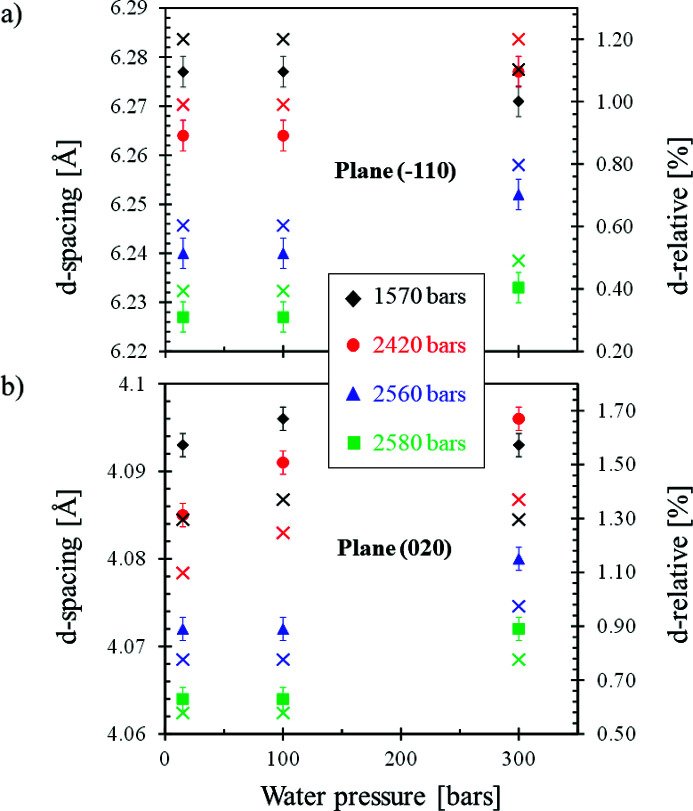
Extracted peak positions from Gaussian fits (filled symbols) and relative variations (cross symbols) with respect to the dry membrane.

The *d*-spacing corresponding to the variation of the peak position induced by the different applied stresses is presented in Fig. 7 ▸. The aforementioned slight influence of the 300 bar water pressure (Fig. 5 ▸), that induces a swelling of the cellulose structure, is therefore confirmed.

It must be pointed out that for Type I dry cellulose, the most significant structural transformations occur above a compressive stress of several tens of kbars. Several studies have been devoted to the influence of applying either a compressive stress (Quesada Cabrera *et al.*, 2011 ▸; Rennhofer *et al.*, 2019 ▸; Shinzawa *et al.*, 2011 ▸; Ryu *et al.*, 1982 ▸) or a hydraulic pressure (Gonçalves *et al.*, 2020 ▸) on the structural properties of cellulose. On one hand, compressive stress induces a densification of the network [packing of the cellulose chains (Rennhofer *et al.*, 2019 ▸)] leading to more dis­ordered amorphous structures (Shinzawa *et al.*, 2011 ▸). This was shown by the decrease of *d*-spacings up to *d* = 0.5 Å for the (200) reflection (Quesada Cabrera *et al.*, 2011 ▸) and by a decrease of crystalline peaks intensity associated with an increase of the amorphous contribution (Shinzawa *et al.*, 2011 ▸). Such a device at wide angles may still be useful, for cellulose or derivatives, to study slow kinetics effects on cellulose degradation or swelling in the presence of water or other solvents (Cantero *et al.*, 2015 ▸; Pintiaux *et al.*, 2019 ▸; Frolich *et al.*, 1928 ▸).

## 4. Conclusion

In conclusion, the present pressure environment enables studying the behavior of soft materials subjected to simultaneous compressive stress and hydrostatic pressure by X-ray scattering. The range of applicable pressures is well adapted for investigating poromechanical coupling in soft porous materials. The performance of the whole setup was tested by synchrotron SAXS and WAXS measurements carried out on a model structure: regenerated cellulose membrane. The investigated membrane undergoes moderate structural modification with compression, in part related to an increase of the structural disorder. Applying hydrostatic pressure leads to a slight swelling of the membrane, mainly compensated by the compression. However, the variations observed by X-ray scattering are sufficient to be measured and analyzed, thus demonstrating the benefit and reliability of our setup. Such a device could then be advantageously used for shedding new light on the link between multiscale structure and fluid transport in materials that deform under moderate pressure.

## 5. Related literature

The following references, not cited in the main body of the paper, have been cited in the supporting information: Kusoglu & Weber (2017 ▸); Rubatat *et al.* (2002 ▸).

## Figures and Tables

**Table 1:** Most common X-ray peak positions and corresponding crystallographic planes for native (Type I) and regenerated (Type II) cellulosic materials The Bragg peaks analyzed in the present work are give in bold.

Type	Reference	Crystallographic plane	*Q* (Å^−1^)
I	Garvey *et al.* (2005 ▸)	(101), 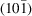 , (101), (002), (040)	1.03, 1.16, 1.42, 1.59, 2.39
I	Poletto *et al.* (2013 ▸)	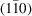 , (110), (200)	1.05, 1.17, 1.57
I	Liu *et al.* (2015 ▸)	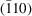 , (110), (200), (004)	1.06, 1.18, 1.61, 2.42
I	Bates *et al.* (2006 ▸)	Amorphous halo	1.52
II	Liu *et al.* (2015 ▸)	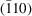 , (110)	**0.87**, **1.45**
II	Li *et al.* (2020 ▸)	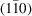 , (110)	**0.88**, **1.46**
II	Jiang *et al.* (2012 ▸)	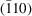 , (002), (110), (012), (020), (103)	**0.87**, 1.22, **1.42**, **1.44**, 1.54, 2.02

## References

[bb1] Bates, S., Zografi, G., Engers, D., Morris, K., Crowley, K. & Newman, A. (2006). *Pharm. Res.* **23**, 2333–2349. 10.1007/s11095-006-9086-2 17021963

[bb2] Briman, I. M., Rébiscoul, D., Diat, O., Zanotti, J.-M., Jollivet, P., Barboux, P. & Gin, S. (2012). *J. Phys. Chem. C*, **116**, 7021–7028.

[bb3] Brooks, N. J., Gauthe, B. L. L. E., Terrill, N. J., Rogers, S. E., Templer, R. H., Ces, O. & Seddon, J. M. (2010). *Rev. Sci. Instrum.* **81**, 064103. 10.1063/1.3449332 20590253

[bb4] Cantero, D. A., Tapia, A. S., Bermejo, M. D. & Cocero, M. J. (2015). *Chem. Eng. J.* **276**, 145–154.

[bb5] Coasne, B., Galarneau, A., Pellenq, R. J. & Di Renzo, F. (2013). *Chem. Soc. Rev.* **42**, 4141–4171. 10.1039/c2cs35384a 23348418

[bb6] Duesing, P., Templer, R. & Seddon, J. (1996). *Rev. Sci. Instrum.* **67**, 4228–4234.

[bb7] French, A. D. (2014). *Cellulose*, **21**, 885–896. 10.1007/s10570-013-0051-z PMC3979627 24729665

[bb8] Frolich, P. K., Spalding, H. B. & Bacon, T. S. (1928). *Ind. Eng. Chem.* **20**, 36–40.

[bb9] Garvey, C. J., Parker, I. H. & Simon, G. P. (2005). *Macromol. Chem. Phys.* **206**, 1568–1575.

[bb10] Gonçalves, S. M., Chávez, D. W. H., de Oliveira, L. M., de Luca Sarantópoulos, C. I. G., de Carvalho, C. W. P., de Melo, N. R. & Rosenthal, A. (2020). *Heliyon*, **6**, e05213. 10.1016/j.heliyon.2020.e05213 PMC7557889 33088965

[bb11] Gubitosi, M., Nosrati, P., Koder Hamid, M., Kuczera, S., Behrens, M. A., Johansson, E. G. & Olsson, U. (2017). *R. Soc. Open Sci.* **4**, 170487. 10.1098/rsos.170487 PMC5579112 28878996

[bb12] Ha, J. & Kim, H.-Y. (2020). *Annu. Rev. Fluid Mech.* **52**, 263–284.

[bb13] Håkansson, K. M. O., Fall, A. B., Lundell, F., Yu, S., Krywka, C., Roth, S. V., Santoro, G., Kvick, M., Prahl Wittberg, L., Wågberg, L. & Söderberg, L. D. (2014). *Nat. Commun.* **5**, 4018. 10.1038/ncomms5018 PMC4059937 24887005

[bb14] He, S., Ye, H., Ma, Y., Guo, L., Gou, Y. & Zhang, P. (2016). *Corros. Sci.* **107**, 21–30.

[bb15] Howell, C. L. (2008). *Understanding wood biodegradation through the characterization of crystalline cellulose nanostructures.* PhD thesis, University of Maine, Maine, USA.

[bb16] Jiang, G., Yuan, Y., Wang, B., Yin, X., Mukuze, K. S., Huang, W., Zhang, Y. & Wang, H. (2012). *Cellulose*, **19**, 1075–1083.

[bb17] Klein, M., Perrin, J.-C., Leclerc, S., Guendouz, L., Dillet, J. & Lottin, O. (2013). *Macromolecules*, **46**, 9259–9269.

[bb18] Kusoglu, A. & Weber, A. Z. (2017). *Chem. Rev.* **117**, 987–1104. 10.1021/acs.chemrev.6b00159 28112903

[bb20] Lehmkühler, F., Schroer, M. A., Markmann, V., Frenzel, L., Möller, J., Lange, H., Grübel, G. & Schulz, F. (2019). *Phys. Chem. Chem. Phys.* **21**, 21349–21354. 10.1039/c9cp04658e 31531471

[bb19] Le Vay, K., Carter, B. M., Watkins, D. W., Dora Tang, T.-Y., Ting, V. P., Cölfen, H., Rambo, R. P., Smith, A. J., Ross Anderson, J. L. & Perriman, A. W. (2020). *J. Am. Chem. Soc.* **142**, 20640–20650. 10.1021/jacs.0c07285 33252237

[bb21] Li, H., Kruteva, M., Mystek, K., Dulle, M., Ji, W., Pettersson, T. & Wågberg, L. (2020). *ACS Nano*, **14**, 6774–6784. 10.1021/acsnano.0c00171 PMC7315634 32383585

[bb22] Liu, Z., Sun, X., Hao, M., Huang, C., Xue, Z. & Mu, T. (2015). *Carbohydr. Polym.* **117**, 99–105. 10.1016/j.carbpol.2014.09.053 25498614

[bb23] MacMinn, C. W., Dufresne, E. R. & Wettlaufer, J. S. (2016). *Phys. Rev. Appl.* **5**, 044020.

[bb24] McMahon, M. I. (2015). *Philos. Trans. R. Soc. A*, **373**, 20130158.

[bb25] Mitra, S., Mukhopadhyay, R., Tsukushi, I. & Ikeda, S. (2001). *J. Phys. Condens. Matter*, **13**, 8455–8465.

[bb26] Nabihah, M. & Shanmugan, S. (2016). *Dig. J. Nanomater. Bio­structures*, **11**, 349–356.

[bb27] Pintiaux, T., Heuls, M., Vandenbossche, V., Murphy, T., Wuhrer, R., Castignolles, P., Gaborieau, M. & Rouilly, A. (2019). *Cellulose*, **26**, 2941–2954.

[bb28] Poletto, M., Pistor, V. & Zattera, A. J. (2013). *Cellulose – Fundamental Aspects*, ch. 2, pp. 45–68. InTech.

[bb29] Pressl, K., Kriechbaum, M., Steinhart, M. & Laggner, P. (1997). *Rev. Sci. Instrum.* **68**, 4588–4592.

[bb30] Quesada Cabrera, R., Meersman, F., McMillan, P. F. & Dmitriev, V. (2011). *Biomacromolecules*, **12**, 2178–2183. 10.1021/bm200253h 21480605

[bb31] Rennhofer, H., Plappert, S. F., Lichtenegger, H. C., Bernstorff, S., Fitzka, M., Nedelec, J.-M. & Liebner, F. W. (2019). *Soft Matter*, **15**, 8372–8380. 10.1039/c9sm01422e 31588953

[bb32] Rosti, M. E., Pramanik, S., Brandt, L. & Mitra, D. (2020). *Soft Matter*, **16**, 939–944. 10.1039/c9sm01678c 31845717

[bb33] Rubatat, L., Rollet, A., Gebel, G. & Diat, O. (2002). *Macromolecules*, **35**, 4050–4055.

[bb34] Ryu, D. D., Lee, S. B., Tassinari, T. & Macy, C. (1982). *Biotechnol. Bioeng.* **24**, 1047–1067. 10.1002/bit.260240503 18546399

[bb35] Shen, G. & Mao, H. K. (2016). *Rep. Prog. Phys.* **80**, 016101. 10.1088/1361-6633/80/1/016101 27873767

[bb36] Shinzawa, H., Awa, K. & Ozaki, Y. (2011). *J. Near Infrared Spectrosc.* **19**, 15–22.

[bb37] Steinhart, M., Kriechbaum, M., Pressl, K., Amenitsch, H., Laggner, P. & Bernstorff, S. (1999). *Rev. Sci. Instrum.* **70**, 1540–1545.

[bb38] Taschin, A., Bartolini, P., Marcelli, A., Righini, R. & Torre, R. (2013). *Faraday Discuss.* **167**, 293–308. 10.1039/c3fd00060e 24640497

[bb39] Teixeira, J., Zanotti, J.-M., Bellissent-Funel, M.-C. & Chen, S.-H. (1997). *Physica B*, **234–236**, 370–374.

[bb40] Winter, R. (2002). *Biochim. Biophys. Acta*, **1595**, 160–184. 10.1016/s0167-4838(01)00342-9 11983394

[bb41] Woenckhaus, J., Köhling, R., Winter, R., Thiyagarajan, P. & Finet, S. (2000). *Rev. Sci. Instrum.* **71**, 3895–3899.

[bb42] Wolanin, J., Giraud, J., Payre, C., Benoit, M., Antonelli, C., Quemener, D., Tahiri, I., Vandamme, M., Zanotti, J.-M. & Plazanet, M. (2021). *Rev. Sci. Instrum.* **92**, 024106. 10.1063/5.0030297 33648089

